# Supplementary material for: Safety and Efficacy of Outpatient Treatments for COVID-19: Real-Life Data from a Regionwide Cohort of High-Risk Patients in Tuscany, Italy (the FEDERATE Cohort)
Source: Viruses. 2023 Feb 5;15(2):438. doi: 10.3390/v15020438 (PMC9967010; doi:10.3390/v15020438)
Supplement: Supplementary file 1 [file viruses-15-00438-s001.zip › viruses-2168053-supplementary.pdf]

Supplementary Table S1. Characteristics of the excluded patients in our cohort.

	**TOTAL (n=140)**	**RMD (n=6)**	**SOT (n=12)**	**MOL (n=61)**	**NRM/r (n=61)**	**p-value**
Sex (n, %)<br>-- Male<br>-- Female<br>Missing	 75 (50.4)<br>63 (49.6)	 3 (50)<br>3 (50)	 (52.9)<br>148 (47.1)	 32 (52.4)<br>29 (57.6)	 32 (42.5)<br>28 (57.5)	0.842
Age (median, IQR)	67.3 (53.8-79.7)	58.5 (43.2-70.5)	75.8 (58.1-81.1)	68.9 (57.3-79.9)	66.9 (50.3-75.6)	0.014
Vaccination (n, %)<br>-- None<br>-- One dose<br>-- Full schedule<br>-- Booster	 16 (11.9)<br>2 (1.5)<br>11 (8.2)<br>105 (78.6)	 0<br>0<br>3 (50.0)<br>3 (50.0)	 3 (25)<br>0<br>2 (16.7)<br>7 (58.3)	 9 (20.4)<br>1 (2.6)<br>4 (21.3)<br>44 (55.7)	 4 (6.9)<br>1 (1.5)<br>2 (3.5)<br>51 (87.9)	 <br><br><br><0.009
Time from symptoms onset to treatment (median days, IQR)	2 (2-3)	3.5 (1-4)	4.5 (3-6)	2 (2-3)	2 (1-3)	0.043
Obese (n, %)	34 (24.5)	1 (16.7)	4 (33.3)	17 (28.3)	12 (19.7)	0.582
CKD (n, %)	13 (9.3)	0	3 (25)	8 (14)	2 (3.3)	0.049
CHD (n, %)	75 (53.6)	3 (50)	10 (83.3)	31 (50.8)	31 (50.8)	0.206
Cancer (n, %)	20 (14.3)	3 (50)	1 (8.3)	7 (11.3)	9 (14.8)	0.075
COPD (n, %)	21 (14.9)	2 (33.3)	2 (16.7)	9 (14.1)	8 (13.1)	0.622
Cognitive impairment (n, %)	10 (7.1)	0	5 (41.7)	0	5 (8.2)	<0.001
Stroke (n, %)	6 (4.1)	1 (16.7)	1 (8.33)	1 (1.6)	3 (4.9)	0.289
Diabetes (n, %)	13 (9.6)	1 (16.7)	3 (25)	3 (4.9)	6 (9.8)	0.158
Immunocompromised (n, %)	37 (26.4)	2 (33.3)	4 (33.3)	10 (16.4)	21 (34.4)	0.147
Current or former smokers (n, %)	5 (3.3)	2 (33.3)	0	0	3 (4.9)	<0.001

Legend: RMD: remdesivir; SOT: sotrovimab; MOL: molnupiravir; NRM/r: nirmatrelvir/ritonavir; IQR: interquartile range; CKD: chronic kidney disease; CHD: chronic heart disease; COPD: chronic obstructive pulmonary disease.

Supplementary Table S2. Analysis of standardized differences showed good balance in baseline covariates between the four groups before and after weighting by inverse probability of treatment weighting.

**Variable**	**Standardized Differences**					
	**SOT vs. RMD**		**MOL vs. RMD**		**NRM/r vs. RMD**	
	**Raw**	**Weighted**	**Raw**	**Weighted**	**Raw**	**Weighted**
Sex	−0.23	0.04	−0.32	−0.15	−0.02	−0.09
Age (y)						
<45	−0.01	0.05	−0.21	−0.09	0.08	−0.09
45–54	0.05	0.05	−0.06	0.01	−0.13	−0.01
55–64	0.1	−0.01	0.13	0.03	0.08	0.25
65–74	−0.01	−0.01	−0.05	0.06	0.11	−0.05
>75	−0.09	−0.07	0.07	−0.02	0.02	−0.04
Vaccinated (2–3 doses)	−0.23	0.01	0.01	−0.18	0.37	−0.07
Time from symptoms onset to treatment	−0.01	0.05	−0.50	−0.06	−0.73	0.11
Obese	0.04	0.03	0.27	0.01	0.14	−0.01
CKD	0.31	0.09	0.09	0.19	−0.11	0.06
CHD	−0.13	−0.06	−0.01	−0.04	−0.16	−0.08

Cancer	−0.06	0.02	−0.41	0.09	−0.01	0.06
COPD	−0.05	−0.05	0.15	−0.07	−0.02	−0.11
Cognitive impairment	0.10	0.11	−0.17	0.04	0.16	−0.01
Diabetes	−0.09	0.07	−0.11	0.01	0.03	0.07
Immunocompromised	0.30	0.035	−0.56	−0.02	0.05	−0.03
Current or former smoker	−0.42	−0.05	−0.27	0.05	−0.41	−0.19

Legend: RMD: remdesivir; SOT: sotrovimab; MOL: molnupiravir; NRM/r: nirmatrelvir/ritonavir; CKD: chronic kidney disease; CHD: chronic heart disease; COPD: chronic obstructive pulmonary disease.
